# Development and Comparison of Predictive Models Based on Different Types of Influencing Factors to Select the Best One for the Prediction of OSAHS Prevalence

**DOI:** 10.3389/fpsyt.2022.892737

**Published:** 2022-07-06

**Authors:** Xin Fan, Mu He, Chang Tong, Xiyi Nie, Yun Zhong, Min Lu

**Affiliations:** ^1^Department of Emergency, Shangrao Hospital Affiliated to Nanchang University, Shangrao People’s Hospital, Shangrao, China; ^2^Department of Otolaryngology-Head and Neck Surgery, The First Affiliated Hospital of Nanchang University, Nanchang, China; ^3^School of Stomatology, Nanchang University, Nanchang, China; ^4^Pediatric Medical School, Nanchang University, Nanchang, China; ^5^Department of Neurosurgery, Yichun People’s Hospital, Yichun, China; ^6^The First Clinical Medical College of Nanchang University, Nanchang, China

**Keywords:** nomogram, blood glucose, blood lipid, risk prediction, obstructive sleep apnea-hypopnea syndrome

## Abstract

**Objective:**

This study aims to retrospectively analyze numerous related clinical data to identify three types of potential influencing factors of obstructive sleep apnea-hypopnea syndrome (OSAHS) for establishing three predictive nomograms, respectively. The best performing one was screened to guide further clinical decision-making.

**Methods:**

Correlation, difference and univariate logistic regression analysis were used to identify the influencing factors of OSAHS. Then these factors are divided into three different types according to the characteristics of the data. Lasso regression was used to filter out three types of factors to construct three nomograms, respectively. Compare the performance of the three nomograms evaluated by C-index, ROC curve and Decision Curve Analysis to select the best one. Two queues were obtained by randomly splitting the whole queue, and similar methods are used to verify the performance of the best nomogram.

**Results:**

In total, 8 influencing factors of OSAHS have been identified and divided into three types. Lasso regression finally determined 6, 3 and 4 factors to construct mixed factors nomogram (MFN), baseline factors nomogram (BAFN) and blood factors nomogram (BLFN), respectively. MFN performed best among the three and also performed well in multiple queues.

**Conclusion:**

Compared with BAFN and BLFN constructed by single-type factors, MFN constructed by six mixed-type factors shows better performance in predicting the risk of OSAHS.

## Introduction

As one of the well-known sleep apnea diseases, the typical pathophysiological feature of obstructive sleep apnea-hypopnea syndrome (OSAHS) is the complete or partial obstruction of the upper airway that occurs repeatedly during sleep, causing intermittent hypoxia, sleep fragmentation, sleep structure disorders, hypercapnia and sympathetic nerve excitation (1, 2). The prevalence of OSAHS has recently escalated globally and exhibits large regional differences, ranging from 9 to 38% in Europe and North America and 8.8–24.2% in China (3, 4). With the prevalence rate of 22% in men and 17% in women, the prevalence of obese people and the elderly is higher (5, 6). A wide range of interactions between OSAHS and many serious diseases such as cardiovascular, neurological and metabolic diseases may cause multiple systems and multiple organ damage (7–11), which will seriously affect the quality of life and life expectancy of OSAHS (12). Currently, the available treatments for OSAHS include holistic therapy, drug therapy (tricyclic drugs and serotonergic drugs), medical devices (including oral appliances and continuous positive airway pressure), and surgical treatment (13). However, the therapeutic effect is limited by the lack of specific drugs and the poor compliance and tolerance of OSAHS patients to mechanical therapy (14).

The current diagnosis of OSAHS relies on polysomnography and clinical symptoms (15, 16). Snoring and nocturia, the main symptoms of OSAHS, can also easily confuse OSAHS with asthma, pharyngitis and other diseases (17–19). In addition, due to the lack of diagnostic equipment, the health care center’s diagnosis of OSAHS is obviously insufficient (20). These are easy to cause misdiagnosis and missed diagnosis. Delays in diagnosis lead to aggravation of multi-system diseases and delayed treatment, which seriously affects patients’ quality of life (21). Therefore, early effective prediction and intervention of OSAHS are essential. The nomogram, a graphical form of a mathematical model that combines biological and clinical variables to determine the probability of a clinical event, has become a reliable tool for many predicting clinical outcomes in cancer and diseases (22). A recent study has attempted to integrate the influencing factors to construct a stable performance nomogram to predict apnea-hypopnea index (AHI) (23). Unfortunately, there is now no research on constructing a nomogram for predicting the probability of OSAHS.

It is now generally believed that OSAHS is a complex, multi-factor and multi-gene disease (24–26), and obesity, age, maleness and metabolic syndrome are now considered risk factors for OSAHS (26–28). Obesity is closely related to OSAHS (29). The incidence of OSAHS is 42–48% in obese males and 8–38% in obese females (30, 31). The incidence of OSAHS among patients with class III obesity [body mass index (BMI) ≥ 40] is 12–30 times greater than in the general population (32). As the most dangerous factor for OSAHS, obesity-related indicators (BMI and more) are closely associated with OSAHS (33). Globally, the prevalence of OSAHS in people aged 65 years and older ranges from 30% to 80% (34), and it ranges from 2% to 4% in middle-aged people (35). In the United States, OSAHS affects 3–7% of men and 2–5% of women in the general population (36). The estimated prevalence of OSAHS in middle-aged men in Hong Kong detected using complete polysomnography was 4.1% (37). The prevalence of OSAHS increases with age, and the prevalence of men is significantly higher than that of women of childbearing age (38), which reveals that the incidence of OSAHS varies with age and gender (39).

As an independent risk factor for abnormal glucose and lipid metabolism (40), OSAHS has also been found in recent studies closely related to blood lipid indicators such as total cholesterol, triglycerides and low-density lipoprotein cholesterol levels (41, 42). Not only that but hyperlipidemia and hyperglycemia are also thought to play a role in OSAHS (26, 43, 44).

This study aims to retrospectively analyze a large number of clinical data closely related to OSAHS to identify potential influencing factors of OSAHS for establishing the best performance predictive nomogram for OSAHS by complex analysis methods. It is hoped that this nomogram can accurately predict the risk of individuals suffering from OSAHS to guide further clinical decision-making.

## Materials and Methods

### Patients

From January 2017 to September 2021, we retrospectively recruited 480 patients undergoing polysomnography (PSG) at the First Affiliated Hospital of Nanchang University and Shangrao People’s Hospital. 119 patients were excluded due to the following exclusion criteria: (1) No complete blood indicators needed for the research, including fasting blood glucose (FBG), glycosylated serum protein (GSP), total cholesterol (TC), triglyceride (TG), high-density lipoprotein (HDL), low-density lipoprotein (LDL), apolipoprotein A-1 (ApoA1), apolipoprotein B (ApoB) and lipoprotein a (Lpa) (*n* = 118); (2) Received OSAHS related treatments (e.g., continuous positive pressure ventilation therapy) (*n* = 0); (3) With severe systemic disease (*n* = 0); (3) Unable to complete body index measurement (*n* = 1); (4) There is missing data (*n* = 0). Finally, 361 subjects recorded a complete prior history, have completed a whole blood index test, standard PSG and body index measurements.

This study was approved by the Ethics Committee of the First Affiliated Hospital of Nanchang University (approval number: 2020-12-139) and complied with the Declaration of Helsinki.

### Body Index Measurements and Prior History Collection

Height (m) and weight (kg) were measured and assigned readings by the professional measurers using the same measuring equipment (automatic height and weight instrument), based on the world health organization (WHO) standard method. BMI was calculated as weight/height^2^.

The professional doctors asked and recorded the patient’s past medical history (hypertension, diabetes, yes or no), age (year), gender (man or woman) and the prior history of smoking and alcohol intake (yes or no).

### Polysomnography for Apnea-Hypopnea Index

All patients used polysomnography based on a laboratory PSG (Respironics, Pittsburgh, United States) to monitor and score respiratory events. Electroencephalogram, electromyogram, electrooculogram, electrocardiogram, nasal cavity and oral airflow, chest and abdominal cavity respiratory activity, snoring, pulse oximetry and other data were simultaneously recorded. All tests started before 10:00 at night and ended after 6:00 am the next day. After the monitoring, the professional and technical personnel will interpret and review according to the 2007 American Sleep Medicine Association Sleep and Related Event Interpretation Standards (45).

AHI obtained through PSG was used to diagnose the patient with OSAHS. According to the standards set by the American Sleep Medicine Association, AHI ≥ 5 is defined as OSAHS (45, 46).

### Variables Screening for Nomogram

We run spearman correlation analysis to explore the correlation between age, BMI, nine blood indicators, and AHI. Furthermore, the results were visualized through the lollipop graph. According to the grouping criteria: AHI ≥ 5, all patients were divided into the OSAHS and non-OSAHS groups. The differences of 16 indicators between the two groups were further compared and continuous variable results were visualized through the box plot. Univariate logistic regression was used to screen for the influencing factors of OSAHS further. The above analysis results are used to integrate to obtain eight influencing factors of OSAHS (age, gender, BMI, FBG, TG, TC, ApoB and HDL).

Finally, six influencing factors were screened from 8 influencing factors by choosing the best Lasso penalization parameter (λ) determined by the smallest k-fold cross-validation with *K* = 10. Similarly, we also identified three influencing factors from all baseline index factors (age, gender and BMI). 4 influencing factors were also identified from all blood index factors (FBG, TG, TC, ApoB and HDL). To compare the performance differences among the nomograms constructed by different types of factors, we use these three groups of influencing factors to construct nomograms to predict the patient’s risk of OSAHS, respectively. The nomogram constructed by six mixed factors was defined as the mixed factors nomogram (MFN). Similarly, baseline factors nomogram (BAFN) of 3 baseline factors and blood factors nomogram (BLFN) of 4 blood factors were defined separately.

### Performance Evaluation of Mixed Factors Nomogram

To repeatedly verify the predictive ability of MFN, 361 patients (whole queue) were randomly divided into training queue (*n* = 253) and test queue (*n* = 108) at a ratio of 7:3. To verify that randomization did not cause a deviation in the distribution of clinical indicators, we used a chi-square test to compare the differences in indicators between the training and test queues. Patients in these three queues were used in the following validation analysis simultaneously. We drew calibration curves to evaluate the accuracy of the nomogram in predicting the risk of OSAHS. Harrell’s concordance index (C-index) was measured to quantify the discrimination performance of the nomogram. The receiver operating characteristic (ROC) curve was used to visualize the predictive performance of the nomogram. We also ran a decision curve analysis (DCA) to determine the clinical benefit of the nomogram by quantifying the net benefits of different threshold probabilities for the three queues of patients.

To fully verify the performance of MFN, we construct 3 bootstrapping datasets by repeatedly randomly selecting 100/500/1,000 samples from all samples based on the bootstrap resampling method. Based on these three bootstrapping datasets, the C-index of MFN was calculated separately (47).

### Performance Comparison of Different Types of Nomograms

In a similar way, the C-indexes based on the three types of nomograms were calculated and compared. The calibration curve, ROC curve, and DCA curve that incorporates the curves of the three types of nomograms are drawn separately to compare the performance differences of these nomograms.

### Statistical Analysis

Depending on the features of the distribution, differences between continuous variables were classified by the student’s *t*-test or Mann–Whitney test and differences between categorical variables were classified by chi-square test or Fisher’s exact test. The correlation between the variables was confirmed by Spearman correlation analysis. Univariate logistic regression was used to screen for the influencing factors of OSAHS further. It is stated that all analysis techniques were provided by the R programming language (version 4.0.3) and SPSS Statistics 22. R packages “ggpubr,” “reshape2,” “readr,” “glmnet,” “rms,” “ROCR,” “riskRegression” and “rmda” were used. Continuous data with a normal distribution are presented as the mean ± standard deviation; those with a skewed distribution are presented as the median [with interquartile range (IQR)]. Categorical data are presented as numbers (%). *P* < 0.05 was thought as statistically significant.

## Results

### Variables Screening for Nomogram

**FIGURE 1:**
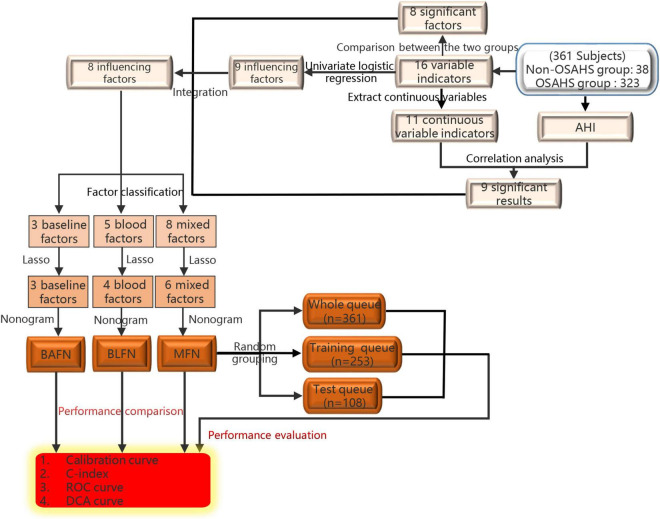
The flow chart of the entire study.

**FIGURE 2:**
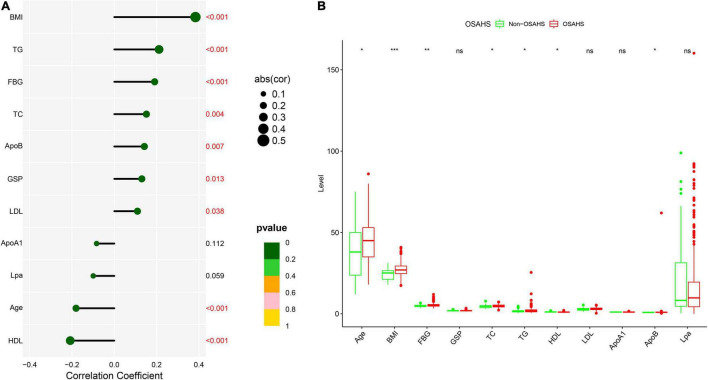
Correlation and difference analysis of 11 clinical continuous indicators. **(A)** Correlation analysis between AHI and 11 continuous indicators. Different sizes and colors of circles represent different correlation coefficients and significance *p*-value, respectively. **(B)** Differences of 11 continuous indicators between OSAHS and non-OSAHS groups. Different symbols are shown at the top of the block diagram. ns: *p* > 0.05; *p < 0.05; **p < 0.01; ***p < 0.001.

**FIGURE 3:**
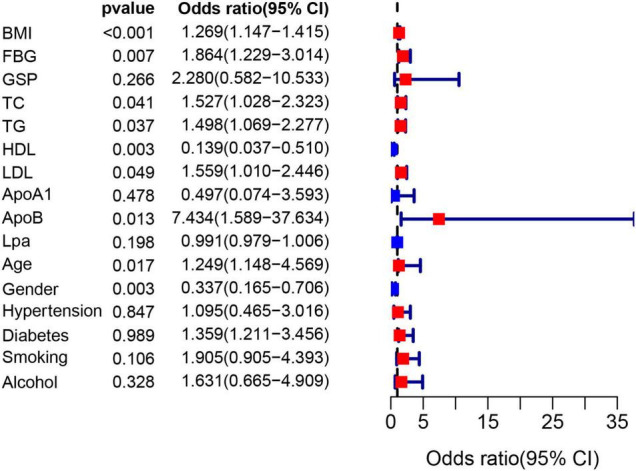
The forest plot shows the univariate logistic regression results of 16 indicators. The red and blue boxes represent the corresponding Odds ratio greater than 1 and less than 1, respectively.

The flow chart of the entire study is shown in Figure 1. Table 1 shows the statistical results of each index of 361 patients. Finally, 38 and 323 patients were divided into non-OSAHS and OSAHS groups, respectively. The lollipop plot showed a positive correlation between BMI/TG/FBG/TC/ApoB/GSP/LDL level and AHI as well as a negative correlation between age/HDL level and AHI (Figure 2A). The results of the differences in 16 indicators and AHI between the OSAHS and non-OSAHS groups are shown in Table 1. The results of 9 indicators (AHI, age, gender, BMI, FBG, TG, TC, ApoB and HDL) show significance. Significantly more men, higher AHI, age, BMI, FBG, TG, TC and ApoB levels, and lower HDL levels were observed in the OSAHS group (Table 1 and Figure 2B). Through univariate Logistic Regression analysis, age, gender, BMI, FBG, TG, TC, ApoB, LDL and HDL were identified as the influencing factors of OSAHS (Table 2 and Figure 3). After integrating the above analysis results, eight variables were identified as the influencing factors of OSAHS (age, gender, BMI, FBG, TG, TC, ApoB and HDL).

**TABLE 1:** Comparison of baseline characteristics, AHI and blood indexes between the non-OSAHS and OSAHS groups.

Variables		Whole queue	Non-OSAHS group	OSAHS group	*p*
		*n* = 361	*n* = 38	*n* = 323	
Age [median (IQR)]		44.00 [33.00, 52.00]	38.00 [23.75, 50.00]	45.00 [35.00, 53.00]	**0.021**
AHI [median (IQR)]		40.20 [16.90, 64.90]	2.40 [1.10, 3.40]	46.80 [21.90, 68.05]	**<0.001**
BMI [median (IQR)]		26.70 [24.51, 29.23]	25.10 [21.21, 26.57]	26.95 [24.71, 29.39]	**<0.001**
FBG [median (IQR)]		5.15 [4.68, 5.67]	5.03 [4.39, 5.22]	5.21 [4.71, 5.75]	**0.005**
GSP [median (IQR)]		1.92 [1.79, 2.09]	1.90 [1.81, 1.97]	1.93 [1.79, 2.09]	0.274
TC [median (IQR)]		4.74 [4.16, 5.32]	4.53 [3.74, 5.08]	4.76 [4.22, 5.40]	**0.044**
TG [median (IQR)]		1.79 [1.21, 2.43]	1.36 [1.19, 1.98]	1.83 [1.21, 2.46]	**0.02**
HDL [median (IQR)]		1.06 [0.91, 1.23]	1.14 [0.99, 1.34]	1.04 [0.91, 1.22]	**0.016**
LDL [median (IQR)]		3.02 [2.51, 3.56]	2.80 [2.19, 3.34]	3.02 [2.54, 3.58]	0.05
ApoA1 [median (IQR)]		1.07 [0.97, 1.19]	1.08 [0.98, 1.22]	1.07 [0.96, 1.19]	0.552
ApoB [median (IQR)]		0.94 [0.80, 1.10]	0.86 [0.71, 1.05]	0.94 [0.81, 1.12]	**0.027**
Lpa [median (IQR)]		9.60 [4.50, 20.50]	8.25 [4.65, 31.38]	9.80 [4.35, 19.50]	0.547
Age [n (%)]	≤35	101 (28.0)	17 (44.7)	84 (26.0)	**0.025**
	>35	260 (72.0)	21 (55.3)	239 (74.0)	
Gender [n (%)]	Man	294 (81.4)	24 (63.2)	270 (83.6)	**0.004**
	Woman	67 (18.6)	14 (36.8)	53 (16.4)	
Hypertension [n (%)]	Non-hypertension	300 (83.1)	32 (84.2)	268 (83.0)	1
	Hypertension	61 (16.9)	6 (15.8)	55 (17.0)	
Diabetes [n (%)]	Non-diabetes	348 (96.4)	38 (100.0)	310 (96.0)	0.424
	Diabetes	13 (3.6)	0 (0.0)	13 (4.0)	
Smoking [n (%)]	Non-smoking	232 (64.3)	29 (76.3)	203 (62.8)	0.144
	Smoking	129 (35.7)	9 (23.7)	120 (37.2)	
Alcohol [n (%)]	Non-alcohol	292 (80.9)	33 (86.8)	259 (80.2)	0.442
	Alcohol	69 (19.1)	5 (13.2)	64 (19.8)	

*p: a value of p < 0.05 was considered significant. OSAHS, obstructive sleep apnea-hypopnea syndrome; IQR, interquartile range; AHI, apnea-hypopnea index; BMI, body mass index; FBG, fasting blood glucose; GSP, glycosylated serum protein; TC, total cholesterol; TG, triglyceride; HDL, high-density lipoprotein; LDL, low-density lipoprotein; ApoA1, apolipoprotein A-1; ApoB, apolipoproteins B; Lpa, lipoprotein a; bold type: significant at p < 0.05.*

**TABLE 2:** Results of univariate Logistic Regression analysis based on 16 variables.

Variable	Beta	Odds ratio (95% CI)	*p*
BMI	0.23844	1.269 (1.147–1.415)	**<0.001**
FBG	0.622465	1.864 (1.229–3.014)	**0.007**
GSP	0.824238	2.280 (0.582–10.533)	0.266
TC	0.423058	1.527 (1.028–2.323)	**0.041**
TG	0.404259	1.498 (1.069–2.277)	**0.037**
HDL	−1.97424	0.139 (0.037–0.510)	**0.003**
LDL	0.444213	1.559 (1.010–2.446)	**0.049**
ApoA1	−0.69882	0.497 (0.074–3.593)	0.478
ApoB	2.006107	7.434 (1.589–37.634)	**0.013**
Lpa	−0.00866	0.991 (0.979–1.006)	0.198
Age	0.834338	1.249 (1.148–4.569)	**0.017**
Gender	−1.08913	0.337 (0.165–0.706)	**0.003**
Hypertension	0.090323	1.095 (0.465–3.016)	0.847
Diabetes	15.46708	1.359 (1.211–3.456)	0.989
Smoking	0.644357	1.905 (0.905–4.393)	0.106
Alcohol	0.489125	1.631 (0.665–4.909)	0.328

*p: a value of p < 0.05 was considered significant. CI, confidence interval; BMI, body mass index; FBG, fasting blood glucose; GSP, glycosylated serum protein; TC, total cholesterol; TG, triglyceride; HDL, high-density lipoprotein; LDL, low-density lipoprotein; ApoA1, apolipoprotein A-1; ApoB, apolipoproteins B; Lpa, Lipoprotein a; bold type: significant at p < 0.05.*

**FIGURE 4:**
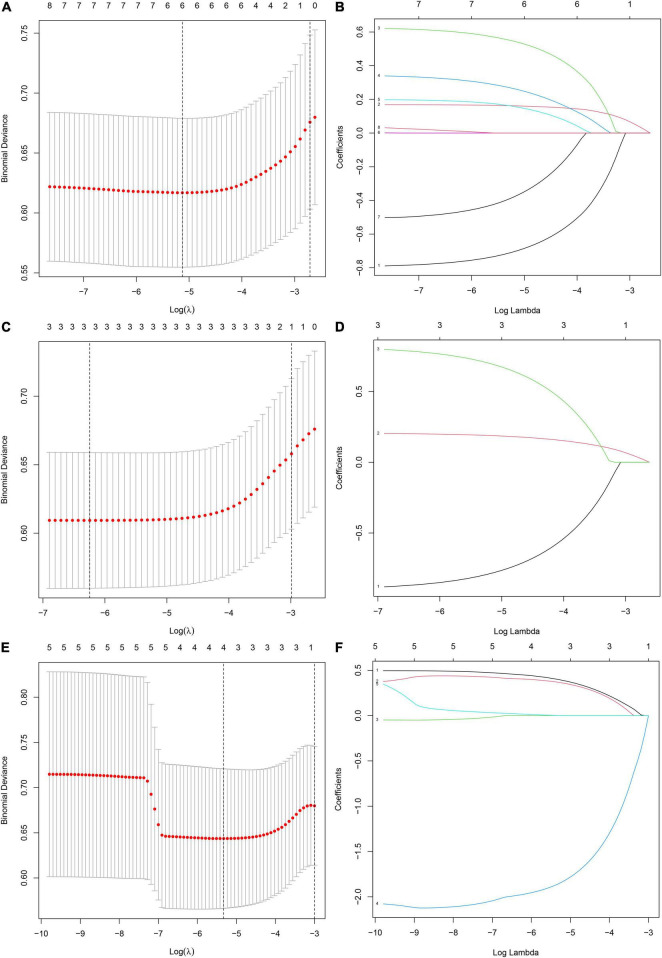
Different types of influencing factors (6 mixed factors, three baseline factors, four blood factors) selection process using lasso regression for nomogram, respectively. **(A,B)** Six mixed factors. **(C,D)** Three baseline factors. **(E,F)** Four blood factors. **(A,C,E)** The optimal parameters (lambda) in 3 LASSO models are selected with 10-fold cross-validation *via* minimum criteria, respectively (59). Draw vertical dashed lines at the optimal value using the minimum criteria and the 1 SE of the minimum criteria (47). **(B,D,F)** Distribution diagrams of LASSO coefficients for three types of factors generated by log (lambda) sequence, respectively (47). Vertical lines are drawn at the best lambda to select 6, 3, and 4 factors with non-zero coefficients, respectively.

**FIGURE 5:**
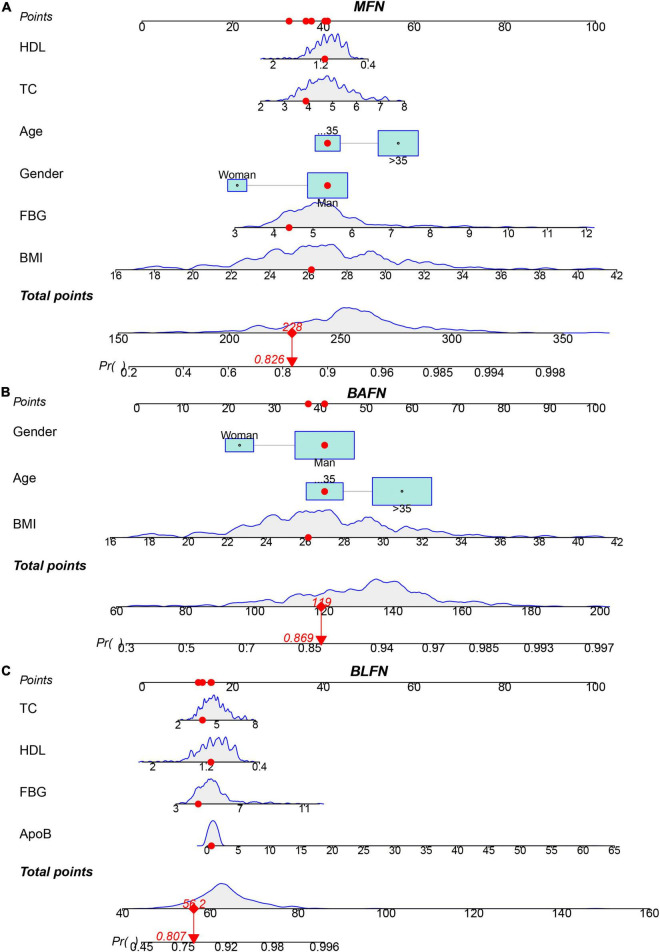
Three types of factors screened by lasso regression were used to construct MFN, BAFN and BLFN, respectively. **(A)** MFN. **(B)** BAFN. **(C)** BLFN.

Eight influencing factors are divided into three types (3 baseline factors: age, gender and BMI; 5 blood factors: FBG, TG, TC, ApoB and HDL; 8 mixed factors: age, gender, BMI, FBG, TG, TC, ApoB and HDL.). To compare the performance differences of the nomograms constructed by different influencing factors, we used three types of factors to run lasso regression analysis to filter the variables to construct the nomogram, respectively. Six mixed factors (age, gender, BMI, FBG, TC and HDL, Figures 4A,B), three baseline factors (age, gender and BMI, Figures 4C,D) and four blood factors (FBG, TC, ApoB and HDL, Figures 4E,F) were selected to construct a MFN (Figure 5A), BAFN (Figure 5B) and BLFN (Figure 5C).

### Performance Comparison of Different Types of Nomograms

**FIGURE 6:**
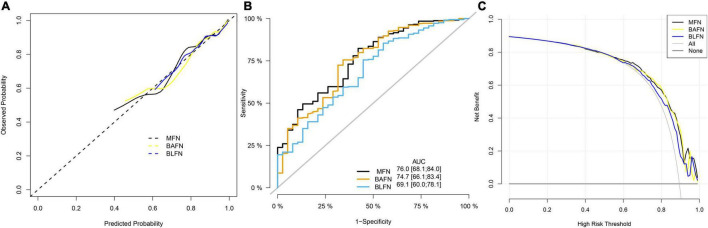
Comparison of the prediction performance of MFN, BAFN and BLFN. **(A)** Calibration curves of mixed results. The x-axis and y-axis represent the risk predicted by the nomogram and the risk diagnosed. The diagonal dashed line represents the perfect prediction of the ideal model (47). The black, yellow and blue solid lines represent the performance of MFN, BAFN and BLFN, respectively, of which a closer fit to the diagonal dotted line represents a better prediction. **(B)** ROC curves of mixed results. The X axis and Y axis represent 1-specificity and sensitivity, respectively. The lower right corner of the figure shows the corresponding AUC values of MFN, BAFN and BLFN, and the 95% confidence interval, respectively. **(C)** DCA curves of mixed results. The y-axis measures the net benefit (47). The thin solid gray line and the thin black solid line represent the assumptions that all patients rely on and do not rely on MFN predictions, respectively. The black, yellow, and blue thick solid lines represent the predictions of MFN, BAFN, and BLFN, respectively.

To verify the better performance of the nomogram constructed by mixed types of factors, including baseline indicators and blood indicators, compared to single type factor, such as baseline indicators or blood indicators, we have constructed three types of nomograms (MFN, BAFN and BLFN). The mixed internal calibration curve shows that the predicted probabilities of the three nomograms are all close to the actual probabilities, indicating that the prediction accuracy of the three nomograms is relatively high (Figure 6A). Compared to BAFN’s C-index (0.747, 95%CI: 0.661–0.834) and BLFN’s C-index (0.691, 95%CI: 0.601–0.782), C-indexes for predicting probability of OSAHS through MFN is the highest (0.761, 95%CI: 0.681–0.840). The mixed ROC curve further supports this conclusion (Figure 6B). These results preliminarily indicate that the prediction performance of MFN is better than the other two types of nomograms. Compared to the benefit threshold probability interval of BAFN (34%–90%) and the benefit threshold probability interval of BLFN (45%–90%), the benefit threshold probability interval of MFN (25%–89%) is the widest.

It can also be seen from the three benefit curves that the overall benefit rate of MFN in the interval is higher than that of the other two types of nomograms (Figure 6C). Based on the results of the above analysis, we can confirm that the prediction performance of the MFN constructed by mixed types of factors is better than the other two nomograms constructed by the single types of factors.

### Performance Evaluation of Mixed Factors Nomogram

**FIGURE 7:**
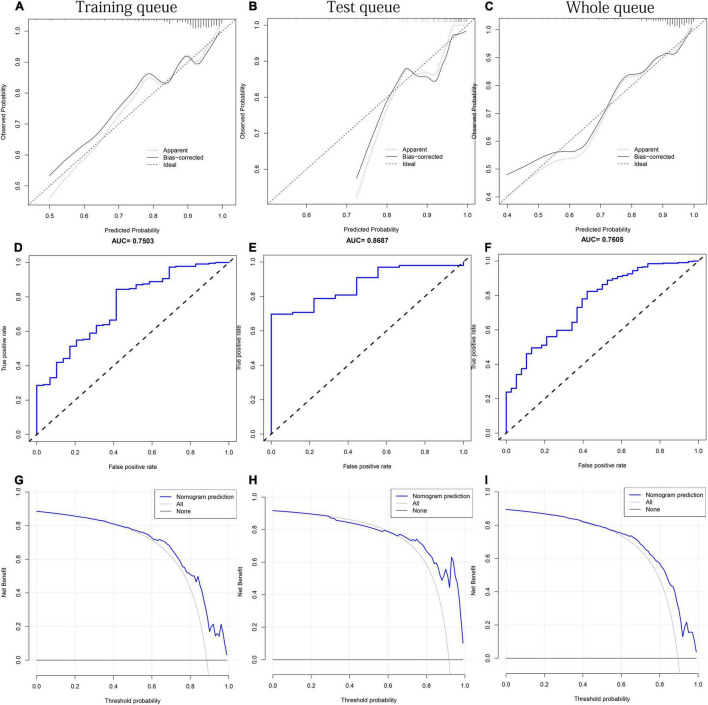
The performance test of MFN prediction in the training, test and the whole queues, respectively. **(A–C)** Calibration curves. The black solid line represents the performance of the nomogram. **(D–F)** ROC curves. The X axis and Y axis represent false positive and true positive rates, respectively. The top of the figure shows the corresponding AUC value. **(G–I)** DCA curves. The blue thick solid lines represent the predictions of MFN.

No significant difference was observed in any clinical indicator between the training and test queues (*p* > 0.05), which shows that the randomization did not cause the deviation of the indicator (Table 3). To fully verify the predictive performance and accuracy of MFN, the c-index, calibration curve, ROC curve and DCA curve based on the data of the three queues were run separately. In the training, test, and whole queues, it was observed that the probability of OSAHS predicted by MFN was in good agreement with the actual probability, indicating that MFN has an accurate predictive ability (Figures 7A–C). C-indexes for predicting probability of OSAHS through MFN calculated using training, test, and whole queue data were 0.750 (95%CI: 0.657–0.844), 0.869 (95%CI: 0.778–0.959), 0.761 (95%CI: 0.681–0.840), respectively. The three queues’ Area Under Curves (AUC) are also shown in the ROC curves (Figures 7D–F), respectively. In addition, the C-index of MFN calculated from 100/500/1,000 resamples is 0.720/0.717/0.718, respectively. The higher c-indexes and AUCs of all queues indicate that MFN has a good prediction accuracy in the probability of OSAHS.

**TABLE 3:** Statistics of indicators for three queues.

Variables		Whole queue	Training queue	Test queue	*p*
		*n* = 361	*n* = 253	*n* = 108	
Age [median (IQR)]		44.00 [33.00, 52.00]	45.00 [33.00, 53.00]	44.00 [33.00, 50.25]	0.258
AHI [median (IQR)]		40.20 [16.90, 64.90]	38.80 [15.40, 63.90]	42.35 [18.42, 68.20]	0.338
BMI [median (IQR)]		26.70 [24.51, 29.23]	26.75 [24.51, 29.40]	26.51 [24.44, 28.93]	0.388
FBG [median (IQR)]		5.15 [4.68, 5.67]	5.21 [4.71, 5.75]	5.11 [4.58, 5.58]	0.172
GSP [median (IQR)]		1.92 [1.79, 2.09]	1.92 [1.79, 2.09]	1.93 [1.81, 2.09]	0.694
TC [median (IQR)]		4.74 [4.16, 5.32]	4.78 [4.18, 5.37]	4.60 [4.14, 5.18]	0.159
TG [median (IQR)]		1.79 [1.21, 2.43]	1.79 [1.24, 2.50]	1.79 [1.15, 2.30]	0.49
HDL [median (IQR)]		1.06 [0.91, 1.23]	1.06 [0.91, 1.24]	1.06 [0.91, 1.19]	0.741
LDL [median (IQR)]		3.02 [2.51, 3.56]	3.07 [2.53, 3.61]	2.91 [2.50, 3.40]	0.3
ApoA1 [median (IQR)]		1.07 [0.97, 1.19]	1.08 [0.97, 1.20]	1.05 [0.97, 1.17]	0.426
ApoB [median (IQR)]		0.94 [0.80, 1.10]	0.96 [0.81, 1.12]	0.90 [0.80, 1.05]	0.11
Lpa [median (IQR)]		9.60 [4.50, 20.50]	9.80 [4.40, 20.50]	9.25 [4.60, 20.47]	0.789
Age [n (%)]	≤35	101 (28.0)	71 (28.1)	30 (27.8)	1
	>35	260 (72.0)	182 (71.9)	78 (72.2)	
Gender [n (%)]	Man	294 (81.4)	205 (81.0)	89 (82.4)	0.872
	Woman	67 (18.6)	48 (19.0)	19 (17.6)	
Hypertension [n (%)]	Non-hypertension	300 (83.1)	208 (82.2)	92 (85.2)	0.592
	Hypertension	61 (16.9)	45 (17.8)	16 (14.8)	
Diabetes [n (%)]	Non-diabetes	348 (96.4)	243 (96.0)	105 (97.2)	0.81
	Diabetes	13 (3.6)	10 (4.0)	3 (2.8)	
Smoking [n (%)]	Non-smoking	232 (64.3)	161 (63.6)	71 (65.7)	0.793
	Smoking	129 (35.7)	92 (36.4)	37 (34.3)	
Alcohol [n (%)]	Non-alcohol	292 (80.9)	207 (81.8)	85 (78.7)	0.587
	Alcohol	69 (19.1)	46 (18.2)	23 (21.3)	

*p: a value of p < 0.05 was considered significant. IQR, interquartile range; AHI, apnea-hypopnea index; BMI, body mass index; FBG, fasting blood glucose; GSP, glycosylated serum protein; TC, total cholesterol; TG, triglyceride; HDL, high-density lipoprotein; LDL, low-density lipoprotein; ApoA1, apolipoprotein A-1; ApoB, apolipoproteins B; Lpa, lipoprotein a.*

It can be seen from the DCA curve of the training queue that the threshold probability of the net benefit of MFN in the training queue ranges from 10% to 90% (Figure 7G). This means that if the threshold probability is between 10% and 90%, using MFN to predict OSAHS is more beneficial than not using MFN prediction. Similar conclusions can also be obtained in the threshold probability interval (58%–92%) of the test queue (Figure 7H) and the threshold probability interval (25%–89%) of the whole queue (Figure 7I). Therefore, the results of the three queues all indicate that the population can benefit from the prediction of the probability of OSAHS by MFN.

## Discussion

This study collected the baseline, prior history, and blood index data of 38 non-OSAHS and 323 OSAHS patients to establish a nomogram to accurately predict the probability of OSAHS so as to conduct individualized and accurate screening of potential OSAHS patients. Through correlation, difference, and univariate logistic regression analysis, eight influencing factors that significantly affect OSAHS were identified. Then divide these eight factors into three different types according to data characteristics. Lasso regression finally determined 6, 3 and 4 factors to construct MFN, BAFN and BLFN, respectively. By comparing the results of the calibration curves, C-index, ROC curve and DCA analysis of the three nomograms, the best predictive performance of MFN was determined. To further thoroughly verify the performance of MFN, the whole research queue was randomly divided into two queues. MFN based on these two queues also showed excellent predictive performance in various validation analyses.

BMI, a gold indicator of obesity, was the strongest positively correlated with AHI in the correlation analysis. The higher age and BMI, and more men 35 years and older in the OSAHS group also were observed in different analyses. Surprisingly, these results are highly consistent with some epidemiological results. For example, a survey found that 9% of women and 24% of men between 30 and 60 years old have experienced sleep apnea, while 2% of women and 4% of men have been diagnosed with OSAHS (48). A study even believed that OSAHS is most common in men over 40 years of age (38), while another study considered that OSAHS was most common in obese middle-aged men (49). Univariate logistic regression further identifies these three factors as risk factors (Odds ratio > 1, *p* < 0.05). These all support the conclusion that previous studies have regarded them as the vital risks factor for OSAHS (26–28, 33, 38, 39).

Obesity is closely related to OSAHS (29), and approximately 40–70% of obese people are diagnosed with OSAHS (50, 51). At present, obesity is considered to affect the progress of OSAHS mainly through weight-dependent and physiology-dependent mechanisms (49). The weight-dependent mechanism believes that the increased fat deposits in the tongue and/or pharynx tissue is too heavy for the reduced muscle tone that often occurs during rapid eye movement REM sleep (52, 53). Airway obstruction, apnea or hypopnea can all be caused by the increased physical weight of these tissues. Lung mechanics is also affected by increased body mass, reducing functional residual volume and tidal volume (54). The physiology-dependent mechanism believes that the physiological components of obesity, including blood sugar control, insulin action and leptin signaling, may lead to respiratory disturbances and increased central sleep apnea by reducing chemical sensitivity (49, 55, 56). The negative impact of aging on the ability to keep the upper airway open may explain the increase in the incidence of OSAHS with age (15).

Previous studies have shown that OSAHS is positively correlated with TG, TC, LDL levels and negatively correlated with HDL levels (41). Studies have also shown that OSAHS patients are accompanied by dyslipidemia, including significantly increased serum levels of TG, TC, very low-density lipoprotein, LDL, and ApoB, and decreased serum levels of HDL and ApoA levels (42). Some studies have also thought that patients with OSAHS often have hyperlipidemia, blood glucose metabolism disorders and diabetes, but the specific mechanism of action between them is still unclear (42, 57). These are consistent with the analysis results of related indicators in our research. Based on these conclusions, logistic regression analysis further identified FBG, TG, TC, ApoB as risk factors (Odds ratio > 1, *p* < 0.05), and HDL as protective factors of OSAHS (Odds ratio < 1, *p* < 0.05).

Ineffective treatment and the serious harm of multiple systems reflect the extreme importance of early predictive diagnosis and early intervention of OSAHS. The nomogram constructs a scoring system based on the scores of each clinical member factor and the total score (58). Doctors can predict the probability of individuals suffering from OSAHS through this convenient scoring system, which caters to the trend of personalized medicine and provides effective guidance for the early prevention of OSAHS (58). To develop a more scientific nomogram similar to a combination of complex factors in the human body, we used multiple types of influencing factors of OSAHS to determine the optimal combination of members through lasso regression. To verify the performance advantages of MFN constructed by mixed-types factors, we also used lasso regression to screen the other two types of single-type influencing factors to construct BAFN and BLFN, respectively. As expected, MFN showed the best adequate discrimination among the three (MFN: AUC = 0.761, 95%CI: 0.681–0.8400; BAFN: 0.747, 95%CI: 0.661–0.834; BLFN: 0.691, 95% CI: 0.601–0.782). MFN also maintains this performance in the two verification queues (training queue: AUC = 0.750, 95%CI: 0.657–0.844; test queue: AUC = 0.869, 95%CI: 0.778–0.959). DCA, a new method that provides insight into clinical consequences and derives net benefits, was used in this study (58). The MFN decision curve of all queues shows that within the threshold probability interval, compared with not using MFN, using MFN to predict OSAHS will increase more clinical benefits (58). By comparing the DCA curves of the three nomograms, MFN was also found to have the widest benefit range (MFN: 25%–89%; BAFN: 34%–90%; BLFN: 45%–90%). There is no doubt that these complex assessments and analyses support MFN as an excellent tool for predicting the risks of OSAHS.

Although MFN performed well in multiple tests, this study still has many limitations. First of all, this study is considered a retrospective study based on existing data, not a prospective one. The influencing factors of OSAHS are determined only based on the results of the outcome indicators, not the specific process, which will also affect the accuracy of the results. Therefore, it is difficult for us to conduct an in-depth discussion on the specific mechanism of the identified influencing factors. Also limited by the shortcomings of retrospective analysis, the clinical variables included in our analysis are limited. For example, the available blood indicators are limited, which is reflected in fewer biochemical indicators and blood cell indicators. Many data of significant influencing factors of OSAHS, such as mechanical obstruction diseases of the upper airway, internal analysis system diseases (hypothyroidism, hypopituitarism, etc.), genetic factors have not been collected in our study. In addition, quantitative indicators such as the Epworth sleepiness score, which can well quantify the severity of OSAHS, were not included in this study. This will negatively affect the construction of the nomogram with more comprehensive variables more scientific prediction performance. Secondly, the limited sample size will also significantly affect the statistical efficiency, which will affect the reliability of the results and affect the performance of the test nomogram in this study. Finally, the excellent performance demonstrated by MFN still needs to be verified in a large number of clinical applications.

## Data Availability Statement

The original contributions presented in the study are included in the article/supplementary material, further inquiries can be directed to the corresponding author/s.

## Author Contributions

XF and ML conceived and designed the study and wrote the manuscript. XF provided contributions to data analysis. XF and YZ prepared the figures. XF, XN, MH, and CT revised the manuscript. All authors contributed to the article and approved the submitted version.

## Conflict of Interest

The authors declare that the research was conducted in the absence of any commercial or financial relationships that could be construed as a potential conflict of interest.

## Publisher’s Note

All claims expressed in this article are solely those of the authors and do not necessarily represent those of their affiliated organizations, or those of the publisher, the editors and the reviewers. Any product that may be evaluated in this article, or claim that may be made by its manufacturer, is not guaranteed or endorsed by the publisher.

## Abbreviations

OSAHS: obstructive sleep apnea-hypopnea syndrome
AHI: apnea-hypopnea index
BMI: body mass index
PSG: polysomnography
FBG: fasting blood glucose
GSP: glycosylated serum protein
TC: total cholesterol
TG: triglyceride
HDL: high-density lipoprotein
LDL: low-density lipoprotein
ApoA1: apolipoprotein A-1
ApoB: apolipoprotein B
Lpa: lipoprotein a
WHO: World Health Organization
MFN: mixed factors nomogram
BAFN: baseline factors nomogram
BLFN: blood factors nomogram
C-index: concordance index
ROC: receiver operating characteristic
DCA: decision curve analysis
IQR: interquartile range
CI: confidence interval.
